# Regulatory Shifts in Plastid Transcription Play a Key Role in Morphological Conversions of Plastids during Plant Development

**DOI:** 10.3389/fpls.2017.00023

**Published:** 2017-01-19

**Authors:** Monique Liebers, Björn Grübler, Fabien Chevalier, Silva Lerbs-Mache, Livia Merendino, Robert Blanvillain, Thomas Pfannschmidt

**Affiliations:** Laboratoire de Physiologie Cellulaire et Végétale, Institut de Biosciences et Biotechnologies de Grenoble, CNRS, CEA, INRA, Université Grenoble AlpesGrenoble, France

**Keywords:** plastids, plastid morphology, photomorphogenesis, plant development, transcription, gene regulation, NEP, PEP

## Abstract

Plastids display a high morphological and functional diversity. Starting from an undifferentiated small proplastid, these plant cell organelles can develop into four major forms: etioplasts in the dark, chloroplasts in green tissues, chromoplasts in colored flowers and fruits and amyloplasts in roots. The various forms are interconvertible into each other depending on tissue context and respective environmental condition. Research of the last two decades uncovered that each plastid type contains its own specific proteome that can be highly different from that of the other types. Composition of these proteomes largely defines the enzymatic functionality of the respective plastid. The vast majority of plastid proteins is encoded in the nucleus and must be imported from the cytosol. However, a subset of proteins of the photosynthetic and gene expression machineries are encoded on the plastid genome and are transcribed by a complex transcriptional apparatus consisting of phage-type nuclear-encoded RNA polymerases and a bacterial-type plastid-encoded RNA polymerase. Both types recognize specific sets of promoters and transcribe partly over-lapping as well as specific sets of genes. Here we summarize the current knowledge about the sequential activity of these plastid RNA polymerases and their relative activities in different types of plastids. Based on published plastid gene expression profiles we hypothesize that each conversion from one plastid type into another is either accompanied or even preceded by significant changes in plastid transcription suggesting that these changes represent important determinants of plastid morphology and protein composition and, hence, the plastid type.

## Introduction

Plastids are cellular organelles that can be found only in plant and algae cells. They are of endosymbiotic origin that traces back to an evolutionary event in which a mitochondriate eukaryote took up a photosynthetically active cyanobacteria-like bacterium and established it as a permanent component of the cell, likely with the help of *Chlamydiae* (Ball et al., 2016). The most prominent benefit for the eukaryotic cell in this process was the gain of photosynthesis and the concomitant switch from a heterotrophic to an autotrophic lifestyle (Hohmann-Marriott and Blankenship, 2011). The establishment of a stable endosymbiosis was, however, not an immediate evolutionary jump but a long-ongoing adaptation process in which the engulfed cyanobacteria-like ancestor has lost slowly most of its genetic information toward the nucleus of the host cell by horizontal gene transfer (Abdallah et al., 2000; Martin et al., 2002; Reyes-Prieto et al., 2007). Only a small, but highly conserved set of genes finally remained encoded in the plastids’ own genome of present plants, the plastome (Bock, 2007; Wicke et al., 2011). The vast majority of the proteome of present-day plant plastids is, therefore, encoded in the nucleus and must be imported from the cytosol (Rolland et al., 2012; Demarsy et al., 2014). Nevertheless, the proper expression of plastid genes is absolutely essential for the build-up of protein complexes involved in plastid gene transcription and translation as well as in metabolic processes such as photosynthesis or fatty acid biosynthesis (Jarvis and Lopez-Juez, 2013; Lyska et al., 2013). All major plastid multi-subunit protein complexes are composed of a patchwork of nuclear and plastid encoded subunits and can be established only by a tight coordination of gene expression between the two genetic compartments (Pogson et al., 2015).

Alongside with these molecular and sub-cellular constraints, the establishment of plastid proteomes is strongly influenced by tissue-dependent and environmental cues. Multicellular, terrestrial plants are comprised of different organs with very divergent tissue organization and function. Plastids in these different tissues display large morphological and functional variations which are tightly connected to the function of the corresponding tissue (Schnepf, 1980; Lopez-Juez and Pyke, 2005). An individual plant, thus, possesses several different plastid types that represent distinct manifestations of the same cell organelle. Interestingly, most of these plastid types can interconvert upon environmentally induced changes in plant and tissue development. These morphological and functional conversions are only possible by corresponding changes in the plastid proteome composition. In this mini-review we focus on the specific changes in plastid gene expression that occur before or during transitions between different plastid types in the course of plant development.

### The Different Plastid Types of Plant Cells

**FIGURE 1:**
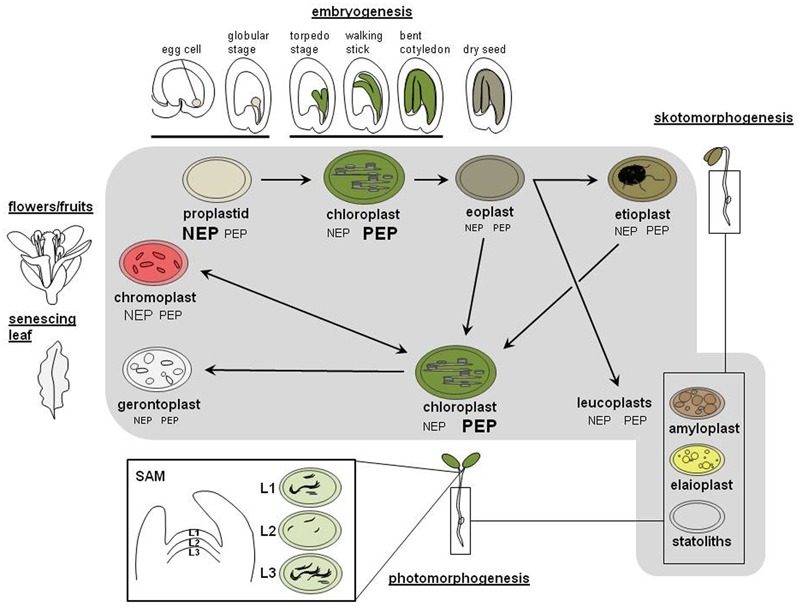
**Transitions between the different plastid types during the plant life cycle.** Important steps in tissue and body development of an angiosperm from fertilization until flower development are depicted in the outer range of the figure using the well characterized life cycle of *Arabidopsis thaliana*. The inner part (gray background) indicates the major plastid types residing in the tissues of the corresponding developmental stage. Arrows indicate type and direction of transition between these plastid types. The inset depicting a cross-cut through a shoot apical meristem (SAM) with its different stages of chloroplast development has been adapted from (Charuvi et al., 2012). L1 – L3 represent different cell layers of SAM containing chloroplasts with different degree of thylakoid membrane development (indicated by rolling lines). Changes in plastid transcriptional apparatus or activity that occur during these transitions are indicated by symbols NEP and PEP. Size of letters represents the relative activities of the two types of RNA polymerases in the respective plastid type. For details see text.

Plant cells cannot generate plastids *de novo* but they gain them by inheritance from their progenitor cell. During division of the mother cell plastids are distributed arbitrarily between daughter cells and multiply afterward, by fission using a prokaryotic-type division apparatus (Osteryoung and Pyke, 2014). The final number of plastids within a cell is cell-type specific and depends on regulatory mechanisms that are far from being understood yet (Cole, 2016). In addition, an individual cell does typically contain only one specific plastid type indicating that plastid development and cell development are interlinked. The various developmental lines and possible conversions between plastid types are subsequently discussed using the life cycle of the angiosperm *Arabidopsis* as a model (**Figure 1**). Because of space constraints detailed species-specific differences or special cases will be not considered here.

In *Arabidopsis* (like in most angiosperms) plastids are inherited maternally as a undifferentiated and small precursor form called proplastid (Pyke, 2007). In other species proplastids might be inherited also by paternal or biparental means. Knowledge in this field is poor and active regulation mechanisms remain to be clarified (Greiner et al., 2015). After fertilization of the egg cell *Arabidopsis* embryos undergo a morphological program typical for angiosperms that eventually ends with dry seeds (Le et al., 2010; compare **Figure 1**, outer circle). Like in many other oilseed crops *Arabidopsis* embryogenesis is characterized by an intermediate photosynthetically active period in which proplastids develop into chloroplasts in a stage-specific manner (Tejos et al., 2010). Chloroplast containing cells are already detected at the globular stage, but are most abundant during 6–12 days after fertilization (Allorent et al., 2013). This phase appears to be important for the fitness of the seed (Allorent et al., 2015). In a subsequent desiccation phase these chloroplasts then de-differentiate into non-photosynthetic, colorless leucoplasts, called eoplasts (Mansfield and Briarty, 1991, 1992). After seed imbibition and germination these eoplasts then re-differentiate into various plastid types depending on tissue context and environmental conditions.

In the dark, seedlings follow a developmental program called skotomorphogenesis (Solymosi and Schoefs, 2010). In cotyledons of such seedlings eoplasts develop into etioplasts while those located in hypocotyl and root develop into different types of colorless leucoplasts that are difficult to distinguish at the morphological level. Etioplasts are characteristic for this developmental program and represent an intermediate stand-by state of chloroplast formation. They do not develop a thylakoid membrane system, but a prolamellar body (PLB) that is composed of regular arrangements of NADPH, the enzyme protochlorophyllide-oxido-reductase (POR), the chlorophyll precursor protochlorophyllide and the thylakoid membrane lipids digalactosyl-diacylglycerol (DGDG) and monogalactosyl-diacylglycerol (MGDG; Bastien et al., 2016). Upon illumination another developmental program called photomorphogenesis is initiated by the phytochrome-mediated photoreceptor network that triggers the expression of many nucleus located genes coding for chloroplast proteins (Arsovski et al., 2012). In parallel, thylakoid membranes begin to form and the light-dependent POR induces chlorophyll biosynthesis within the PLB. Chloroplast biogenesis then is usually completed after just 6–24 h. If seeds germinate directly in light the skotomorphogenic program is skipped and the eoplasts within the cotyledons differentiate directly into chloroplasts. Whether or not proplastids and eoplasts represent fully equivalent developmental stages remain to be elucidated. Studies on the transition of de-differentiated desiccoplasts into etio- or chloroplasts after rehydration and illumination in the poikilochlorophyllous plant *Xerophyta humilis* may provide novel clues for the understanding of proplastid/eoplast-to-chloroplast transitions (Solymosi et al., 2013).

During primary leaf formation chloroplasts originate directly from proplastids present in the shoot apical meristem (SAM; Charuvi et al., 2012). Fully developed chloroplasts in green parts of plants multiply then by fission until they reach the cell-type specific number. This, however, accounts mainly for the mesophyll tissue while in the epidermis a likely tissue-specific program leads to a differential development of chloroplasts. Guard cells were reported to display high numbers of fully developed chloroplasts while pavement cells contain rather low numbers of relatively small chloroplasts (around half the size of those in mesophyll cells) that may contain reduced levels of chlorophyll (Barton et al., 2016). In reproductive organs such as fruits or flowers chloroplasts usually transform into chromoplasts as part of maturation or developmental programs. In senescing tissues the valuable resources of chloroplasts, notably the nitrogen bound in chlorophylls and photosynthesis proteins such as RubisCO, are reallocated and the plastids turn into gerontoplasts, the aging form of plastids.

In hypocotyls and roots of growing seedlings eoplasts usually develop into a number of colorless plastids commonly summarized under the term leucoplasts. This group comprises amyloplasts, statoliths, and elaioplasts (**Figure 1** and **Table 1**) and, in later stages, may develop also in other parts of the plant. These colorless plastids do develop even if the tissues are exposed to light. This strongly suggests that the transition from proplastids/eoplasts into chloroplasts is actively inhibited in these tissues, likely by internal factors. Recent studies have demonstrated that the developmental block of chloroplast development in *Arabidopsis* roots can be released either genetically or by external hormone treatment (Kobayashi et al., 2012) supporting the view of an active inhibition in chloroplast biogenesis in these tissues. Release of such an inhibition represents not only an artificial effect but does occur also under physiological conditions as some studies reported the presence of fully developed chloroplasts in *Arabidopsis* hypocotyls (Jin et al., 2001; Hermkes et al., 2011). These chloroplasts were found to be involved in phototropic responses suggesting that they play a defined physiological role (Jin et al., 2001). Studying the mechanisms that control this eoplast-chloroplast transition could help to understand principle steps of early chloroplast biogenesis and to identify novel regulatory factors of plastid transitions (Chiang et al., 2012).

**Table 1:** Summary of major plastid types in plant cells.

Plastid type	Tissue appearance	Morphological characteristic	Main function	Remarks	Reference
Proplastids, Eoplasts	Germ cells, embryonic and meristematic tissues	Small with low internal differentiation	Transmission of plastids between cells and generations	Terminological definition in different reports can be ambiguous	Pyke, 2007
Etioplasts	Cotyledons of dark-grown seedlings	Prolamellar body (PLB)	Stand-by state for chloroplast biogenesis		Solymosi and Schoefs, 2010
Chloroplasts	All photo-synthetically active tissues, appearance in hypocotyls and roots under certain conditions possible	Thylakoid membrane system	Photosynthesis, reduction of nitrogen and sulfur, biosyntheses of metabolites	Structural and functional variation depending on photosynthesis type (e.g., C3/C4, CAM)	Jarvis and Lopez-Juez, 2013
Chromoplast	Fruits, flowers, roots, but also formerly green tissues	Strong carotenoid synthesis	Pigment storage, tissue coloration	Internal structures may vary with degree of coloration	Egea et al., 2010
Amyloplasts	Roots and non-green storage tissues	Huge, starch grains for long-term storage	Energy storage	Serve as statoliths in gravi-perception of root columella cells	Pyke, 2007
Elaioplasts	Specialized cells, e.g., tapetal cells of anthers	High amounts of plastoglobuli	Lipid storage for pollen wall		Ting et al., 1998

*Typical plastid types found in vascular plants are listed. Leucoplasts are not included as they represent a group of plastids (summarizing all non-green plastids lacking pigments including amyloplasts) rather than defining a specific plastid type. The group of leucoplasts contains also other less prominent plastid types that are not well investigated and not discussed in this review such as root plastids and proteinoplasts. For more information on these specialized plastid forms readers are referred to corresponding reviews (Schnepf, 1980; Pyke, 2007).*

### Shifts in Plastid Transcription during Morphological Transitions of Plastids

The different types of plastids mentioned above perform very different functions that are highly specific for the tissue in which they reside (Pyke, 2007) (**Table 1**). Despite their morphological and functional diversity they all contain the same genome (Bock, 2007). However, their strong functional diversity implies a specific enzymatic configuration for each plastid type. This requires a controlled adjustment in the expression of both plastid and nuclear genes encoding the proteins for each of these specific organelle manifestations. Here, we focus on the adjustment of plastid gene expression.

Molecular and genetic studies uncovered that transcription of plastid genes is performed by two different types of RNA polymerases. One type is comprised by two single-subunit phage-type RNA polymerases encoded by two different nuclear genes (nuclear-encoded RNA polymerases, NEP). These proteins are targeted either only to the plastid (RpoTp) or dually to plastids and mitochondria (RpoTmp). The other type of RNA polymerase is a multi-subunit enzyme of prokaryotic type with four basic subunits encoded in the plastid genome (RpoA, RpoB, RpoC1, and RpoC2; plastid-encoded RNA polymerase, PEP). For promoter recognition this enzyme complex is dependent on the interaction with sigma factors (called Sig1 – Sig6 in *Arabidopsis*) that are encoded in the nucleus (Toyoshima et al., 2005; Schweer et al., 2010; Lerbs-Mache, 2011; Borner et al., 2015; Pfannschmidt et al., 2015). The two types of RNA polymerase activities utilize different promoters and depending on their respective promoter structure the genes on the plastid genome can be categorized into three different classes. Class I comprises genes possessing only PEP promoters (only photosynthesis genes). Class II covers genes that have both NEP and PEP promoters (most other genes including genes for the ATP synthase and many components of the gene expression system). Class III represents genes with NEP promoters only and comprises *ycf2* (encoding a still unknown protein), *accD* (encoding the β-carboxyltransferase subunit of the acetyl CoA carboxylase) and the *rpoBC_1_C_2_* operon (Liere et al., 2011). This diversity of promoter structures and the multiplicity of transcriptional components (see also below) represent a prerequisite for efficient transcriptional regulation during plastid conversion where plastid housekeeping genes are preferentially transcribed by NEP and photosynthesis related genes are transcribed by PEP (Allison et al., 1996; Hajdukiewicz et al., 1997).

We propose that targeted changes in plastid transcription, mostly by controlling the relative activities of NEP and PEP enzymes, impact the establishment of the plastid proteome and, therefore, represent key determinants for the transitions between the different plastid types.

#### Proplastid/Eoplast-Chloroplast Transition

Proplastids can be found only in meristematic cells of plants and in *in vitro* cultured cells. Isolation of proplastids from meristematic cells is technically not feasible. However, as meristematic cells give rise to various plant organs, proplastids might be considered as starting point for differentiation-dependent plastid conversion. Also, plastid gene expression in proplastids and after controlled conversion of proplastids into amyloplasts has been analyzed using *in vitro* cultured cells [(Sakai et al., 1992), see below]. These early experiments already showed that such plastid conversion is accompanied by changes in plastid transcriptional activity.

Proplastid/eoplast-chloroplast conversion-associated changes in plastid gene expression patterns have been characterized in detail during *Arabidopsis* seed formation and germination (Demarsy et al., 2012; Allorent et al., 2013). Although slight increases of NEP transcribed mRNAs were observed in this transition, the predominant changes concern remarkable increases of mRNAs of photosynthesis related proteins. If proplastid/eoplast-chloroplast conversion is prevented by deletion of plastid *rpo* genes, colorless plastids of 2–5 μm length are formed that might be considered as a genetically induced type of leucoplasts (Allison et al., 1996; De Santis-MacIossek et al., 1999). Thus, establishment of the correct NEP/PEP configuration and their relative activities at a given developmental stage is absolutely essential for successful chloroplast differentiation.

#### Etioplast-Chloroplast Transition

Etioplasts and their light-induced transition to chloroplasts are well studied in numerous dicotyledonous and monocotyledonous species. Most striking is the very rapid development of thylakoid membranes, increase in chlorophyll content and construction of the photosynthetic apparatus that requires both a massive import of nuclear encoded plastid proteins and high expression of plastid-encoded genes (Lonosky et al., 2004; von Zychlinski et al., 2005; Philippar et al., 2007; Pudelski et al., 2009; Majeran et al., 2010; Ploscher et al., 2011). Etioplasts display just a basic transcriptional activity and accumulate photosynthesis transcripts only to very low levels. Shifting dark-grown seedlings to light, however, rapidly induce a plastome-wide transcript accumulation of photosynthesis genes reaching a maximum level after 10–44 h mRNA levels followed by decrease to approximate pre-illumination levels (Rodermel and Bogorad, 1985). The initial increase in mRNA is followed by subsequent translation of the corresponding proteins (Kanervo et al., 2008). It should be noted that tissue-specific gene expression analyses distinguishing epidermal and mesophyll tissues were never reported and that the results in all studies to date, thus, represent a mixture of both cell types. This is critical with respect to the notion that recent studies suggest a specific sensor function for epidermal chloroplasts (Virdi et al., 2015, 2016). Targeted research on this special type of chloroplasts will be required in order to understand their detailed physiological function.

The light-dependent activation of plastid gene expression during etioplast-chloroplast conversion includes post-translational modifications such as phosphorylation of PEP subunits and sigma factors (Tiller and Link, 1993) and a restructuring of the PEP complex. While in etiolated mustard seedlings PEP was found to exist in its prokaryotic composition (α2, β, β′, β″ subunits), a much larger PEP complex with many additional subunits was purified from fully developed chloroplasts. Studies on intermediate plastids isolated from seedlings illuminated for just 16 h identified both complexes to around equal activities suggesting a light-induced conversion between these two plastid RNA polymerase complexes (Pfannschmidt and Link, 1994). Detailed mass spectrometry analyses identified these subunits and a set of conserved PEP-associated proteins (PAPs) could be defined (Pfannschmidt et al., 2000; Suzuki et al., 2004; Pfalz et al., 2006; Steiner et al., 2011).

PEP-associated proteins are all nuclear encoded and are rapidly light-induced during etioplast-chloroplast transition yielding the observed PEP restructuring (Yagi et al., 2012). Genetic inactivation of any of these PAPs in *Arabidopsis*, maize or rice results in a block of proper chloroplast development and ends up in albinoic phenotypes suggesting that *pap* gene expression and/or subsequent PEP re-structuring represent essential steps in early chloroplast biogenesis. Evolutionary presence of *pap* genes appears to be restricted to terrestrial plants and ferns suggesting that their appearance is connected to the conquest of land (Pfalz and Pfannschmidt, 2013). These genes, thus, likely represent an evolutionary indicator for the development of chloroplast-containing multi-cellular plants (de Vries et al., 2016).

Plastid gene expression changes during etioplast to chloroplast conversion were also analyzed in the monocotyledonous plant maize. In monocotyledons, leaf development is initiated at a basal meristem resulting in a gradient of chloroplast development from the bottom to the tip (Baumgartner et al., 1989, 1993; Hess et al., 1993). This gradient has been used extensively as a model for chloroplast biogenesis. About 51 plastid genes were found to be at least two times higher expressed in tips than in the leaf base (Cahoon et al., 2008). It is, however, still debated how far this plastid developmental gradient reflects the corresponding situation (proplastid-to-chloroplast conversion) in dicotyledonous plants.

#### Chloroplast-Chromoplast Transition

Chromoplasts mainly develop from chloroplasts in formerly green plant tissues e.g., during fruit ripening or flower development. They can also develop directly from proplastids or amyloplast depending on species and tissue (Egea et al., 2010). Plastid gene expression during conversion of green chloroplasts toward red chromoplasts has been characterized in detail during tomato fruit ripening. In contrast to the rapid etioplast-chloroplast transition in cotyledons, the chloroplast-chromoplast transition in tomato fruits requires several days or even weeks allowing transcript analyses of various intermediary stages. These studies uncovered both systemic and gene-specific effects (Kahlau and Bock, 2008). Most important, green tomato fruits displayed a dramatic reduction in chloroplast transcripts compared to green leaves from the same plant. This indicates that the fruit developmental program provides a dominant repressive impact on plastid transcriptional activities even before ripening effects became visible. This may prevent the unnecessary production of photosynthesis proteins in the tomato fruit already in early stages of ripening.

In contrast, changes in plastid gene expression in the subsequent stages (turning, light red, red) remained relatively subtle suggesting that the chloroplast-chromoplast conversion itself is not accompanied by major changes in plastid gene expression. An exception was observed for the *accD* gene that displayed a targeted accumulation at both, transcript and protein levels. Accumulation of the protein AccD as part of the fatty acid biosynthesis complex may allow the accumulation of lipids necessary for storage of carotenoids produced during fruit ripening. *AccD* gene expression requires at least a low level of expression of genes involved in transcription/translation. Indeed, the repression by the fruit developmental program was stronger for photosynthesis genes than for genetic system genes (Kahlau and Bock, 2008) suggesting that low levels of plastid gene expression activity may remain. These remaining activities may be directed to the observed targeted *accD* gene expression.

#### Proplastid-Amyloplast Transition

Amyloplasts are the plastids of storage organ tissues and roots and typically contain high amounts of starch. Systematic gene expression studies in this plastid type were done using potato tubers (Brosch et al., 2007; Valkov et al., 2009). When compared to leaf chloroplasts tuber amyloplasts displayed very low levels of gene expression in terms of transcriptional rate, transcript accumulation and maturation as well as ribosome association of mRNAs and translation. Both, NEP and PEP enzymes are present, but run-on transcription experiments revealed very low transcriptional rates of both enzyme activities. Interestingly, like in chromoplasts *accD* expression appeared to be an exception. It displayed relatively stable transcript levels and ribosome association (Valkov et al., 2009) thus confirming the importance of AccD for the maintenance of plastids regardless of their morphological type. In addition, trans-plastomic inactivation of the plastid *accD* gene in tobacco revealed to be impossible (Kode et al., 2005).

Tissue cultures of tobacco bright-yellow (BY)-2 cells represent another test system to study amyloplasts (Miyazawa et al., 1999; Enami et al., 2011). In presence of cytokinin these cells develop amyloplasts from proplastids. Microarray analysis of the transcriptome did not reveal specific changes between the two plastid types, including the *accD* gene. Interestingly, inhibitors of plastid transcription or translation blocked the hormone-induced differentiation of amyloplasts indicating signaling of plastid gene expression to the hormone-induced plastid developmental pathway. This specific retrograde signaling pathway seems to act *via* intermediates of tetrapyrrole biosynthesis, i.e., haem (Enami et al., 2011).

## Conclusion and Perspectives

In all plastid conversions investigated so far, changes in the plastid transcriptional apparatus and/or transcriptional activity either accompany or even precede the transition. Proper control of plastome transcription, thus, appears to be an important determinant for these developmental steps. Future research will focus on the identification of regulators that may serve as master switches of plastid development in response to internal and external cues (Lopez-Juez, 2007). In addition, more detailed studies on gene expression in proplastids or eoplasts may be highly informative for understanding the molecular regulation of plastid development especially in their initial steps.

## Author Contributions

ML contributed a figure, contributed to the manuscript text, read, and approved the final version. BG contributed to the manuscript text, read, and approved the final version. FC contributed to the manuscript text, read, and approved the final version. SL-M contributed to the manuscript text, read, and approved the final version. LM contributed to the manuscript text, read, and approved the final version. RB contributed to the manuscript text, read, and approved the final version. TP developed manuscript idea, wrote the manuscript with the help of all co-authors.

## Conflict of Interest Statement

The authors declare that the research was conducted in the absence of any commercial or financial relationships that could be construed as a potential conflict of interest.
